# Changes of Active Substances in *Ganoderma lucidum* during Different Growth Periods and Analysis of Their Molecular Mechanism

**DOI:** 10.3390/molecules29112591

**Published:** 2024-05-31

**Authors:** Xusheng Gao, Huimin Huo, Haiying Bao, Jialu Wang, Dan Gao

**Affiliations:** 1 Key Laboratory of Edible Fungi Resources and Utilization, College of Traditional Chinese Medicine, Ministry of Agriculture and Rural Affairs, Jilin Agricultural University, Changchun 130118, China; gaoxusheng@o.cnu.ac.kr (X.G.); huohuimin1216@163.com (H.H.); 2 Institute of Chinese Materia Medica, China Academy of Chinese Medical Sciences, Beijing 100700, China; jialumarco2019@outlook.com

**Keywords:** *Ganoderma lucidum*, metabolomics, proteomics, Ganoderenic acids

## Abstract

*Ganoderma lucidum*, renowned as an essential edible and medicinal mushroom in China, remains shrouded in limited understanding concerning the intrinsic mechanisms governing the accumulation of active components and potential protein expression across its diverse developmental stages. Accordingly, this study employed a meticulous integration of metabolomics and proteomics techniques to scrutinize the dynamic alterations in metabolite accumulation and protein expression in *G. lucidum* throughout its growth phases. The metabolomics analysis unveiled elevated levels of triterpenoids, steroids, and polyphenolic compounds during the budding stage (BS) of mushroom growth, with prominent compounds including Diplazium and Ganoderenic acids E, H, and I, alongside key steroids such as cholesterol and 4,4-dimethyl-5alpha-cholesta-8,14,24-trien-3beta-ol. Additionally, nutrients such as polysaccharides, flavonoids, and purines exhibited heightened presence during the maturation stage (FS) of ascospores. Proteomic scrutiny demonstrated the modulation of triterpenoid synthesis by the CYP450, HMGR, HMGS, and ERG protein families, all exhibiting a decline as *G*. *lucidum* progressed, except for the ARE family, which displayed an upward trajectory. Therefore, BS is recommended as the best harvesting period for *G. lucidum*. This investigation contributes novel insights into the holistic exploitation of *G. lucidum*.

## 1. Introduction

*Ganoderma lucidum* is an essential edible and medicinal mushroom in China [1,2,3,4]. *G. lucidum*, also referred to as “lingzhi” in Chinese, “reishi” in Japanese, and “Yeongji” in Korean, exhibits a wide range of colors and shapes. In the literature on traditional Chinese medicine (TCM), there are six types of chi: purple, white, red, black, yellow, and blue. *G. lucidum* belongs to the red chi [5,6]. *G. lucidum*, as a functional food with the ability to enhance immunity and anti-aging, is quite popular in China and has been developed in a series of products such as tea, powder, oral liquid, and so on, and is often used to make soups in the southern part of China [1]. Modern pharmacological studies have demonstrated the therapeutic potential of *G. lucidum* in the treatment of a range of chronic diseases. These conditions include liver disease, nephritis, hypertension, arthritis, insomnia, bronchitis, asthma, diabetes, and cancer. The global *G. lucidum* industry has the potential to generate approximately US$ 7.5 billion annually. However, owing to the inadequate availability of wild *G. lucidum*, meeting the growing consumer demand has become a challenge. Consequently, several countries have shifted their focus to cultivating *G. lucidum* to meet market needs. China is a prominent country in this industry and currently cultivates nearly 150 different strains of *G. lucidum*. Most countries can ensure a more consistent supply of fungus by relying on cultivation methods. Bioactive compounds present in *G. lucidum* have shown promising results in both preclinical and clinical studies. The diverse range of medicinal properties exhibited by *G. lucidum* renders it a valuable candidate for developing therapeutic interventions for various health conditions [7,8]. With the advancement of TCM and modern scientific techniques, over 700 compounds have been isolated from *G. lucidum*, including polysaccharides, triterpenoids, sterols, alkaloids, amino acid peptides, and furan derivatives. This significant array of compounds has the potential for development in healthcare, functional foods, and medicine. Unraveling the complex interplay between nutrient accumulation and compositional shifts throughout fruiting body development in *G. lucidum* is crucial for enhancing its quality through meticulous breeding strategies. However, such intricate processes remain largely unknown in most *G. lucidum* cultivars.

As of right now, the mature fruiting bodies and spores of *G. lucidum* are where the active components that are collected and used come from. Notably, when mushrooms age, the amounts of soluble polysaccharides and triterpenes in fruiting bodies may not always rise. Notably, the highest concentrations of ganoderic acid A, ganoderic acid D, and ganodermatriol were observed during the early growth stages [9]. According to Toshinori Nakagawa’s research, there are notable alterations in the polysaccharides and triterpenes of *G. lucidum* during its growth. Specifically, there are elevated levels of triterpenes and sterols before the fruiting body matures, particularly during the stipe elongation stage or the early stages of pileus formation [10]. Ren et al. demonstrated that *G. lucidum* exhibits variations in total phenol, flavonoid, ganoderic acid, and polysaccharide content during the various growth periods [11]. However, the specific composition, type, and dynamics of *G. lucidum*’s active ingredients throughout various developmental stages are currently not comprehensively understood. This knowledge would be valuable for optimizing the utilization of *G. lucidum* at the various growth stages.

Metabolomics offers a comprehensive approach to elucidating the intricate chemical composition of *G. lucidum* [12]. Xia et al. used a metabolomics approach and determined that ganoderic acid distribution was higher at the bottom of the cap mediostratum layer and in the stipe shell and context layers at the young bud stage, but did not reveal other material changes and molecular mechanisms [13]. Proteomics, which is the study of proteins, is a notable method for studying complex biological systems, as it offers insights into the functions and metabolic pathways of proteins [14,15]. Recent advancements in rapid protein identification technologies have facilitated large-scale proteomic analysis [16]. Using this methodology, numerous proteomic studies have been conducted on fungi, such as *Termitomyces heimii*, *Sparassis crispa*, *Hericium erinaceum*, *Arthrobotrys oligospora*, *Metarhizium acridum*, *Agrocybe aegerita*, and *Cordyceps militaris*. Strategies that incorporate metabolomics and proteomics are frequently used to elucidate the extent of proteomic functions and metabolic pathways. Therefore, metabolomic and proteome profiling has been employed to characterize the biochemical pathways and nutritional properties of *G. lucidum* across time in order to explore the dynamic changes in *G. lucidum* during development. *Dictyophora indusiata* benefited greatly from the use of this integrative analysis. For example, Wang et al. used proteomic and metabolomic approaches to reveal the underlying causes of morphological changes in Dictyostelium, in which glycerophospholipids were hydrolyzed and decreased dramatically during the maturation of its fruiting bodies, degradation of dextran was upregulated, degradation and synthesis of chitin were simultaneously enhanced, and proteins were predominantly catabolized [17].

The current work employed a combination of proteomics and metabolomics to thoroughly examine the changes in protein expression and metabolite composition in *G. lucidum* at different growth stages. Gaining further insight into the molecular processes driving these alterations was the main goal of our work. Furthermore, our goal was to pinpoint the major regulatory routes that *G. lucidum*’s morphological changes throughout growth are attributed to. By establishing a theoretical framework, this study improves the productivity and quality of *G. lucidum* by identifying the ideal time to harvest it. To the best of our knowledge, this is the first multi-omics-based comprehensive investigation of the molecular pathways underlying growth variations in *G. lucidum*.

## 2. Results

### 2.1. Changes in Bioactive Nutrient of G. lucidum at Various Growth Periods

**Figure 1:**
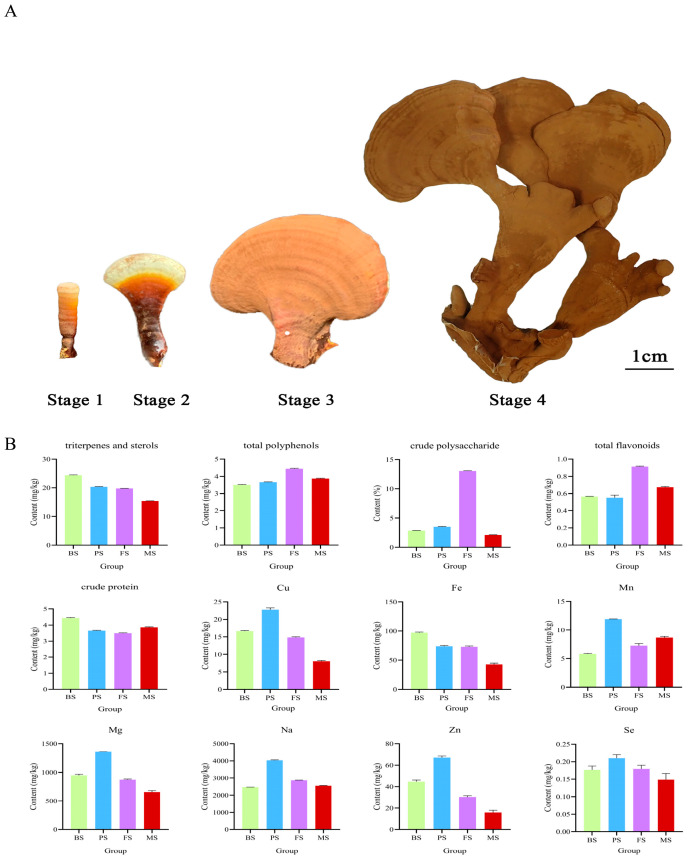
Phenotypes of *G. lucidum* at different growth periods and results of nutrient composition and micro-and macro-elements. (**A**) Phenotypes of *G. Lucidum* at different growth periods: Stage 1 Bud Stage (BS), Stage 2 Opening Stage (PS), Stage 3 Fruit Maturity Stage (FS), Stage 4 Maturation Of Spores (MS) (from left to right); (**B**) Result of nutrient composition and micro- and macro-elements.

The phenotypes of *G. lucidum* at different growth periods are shown in Figure 1A, exhibiting substantial differences. The results of the nutrient and active ingredients analyses showed a gradual increase in the levels of crude polysaccharides, total flavonoids, and total phenols during the growth cycle, with the highest values observed in the FS. Moreover, we observed that the total protein, triterpenoid, and sterol contents were highest in the BS growth stage and exhibited a gradual decline throughout the growth process. Interestingly, in PS, a significant enrichment of macronutrients and trace elements was observed. Changes in the nutrient composition and levels of active substances occurred as the substrate underwent development and maturation (Figure 1B).

Amino acid trends were further investigated and, surprisingly, all amino acids showed a decreasing trend, except for Met, which showed an increasing trend and was the highest at the MS and BS periods (Table 1).

### 2.2. Metabolic Characteristics of G. lucidum at Various Growth Periods

**Figure 2:**
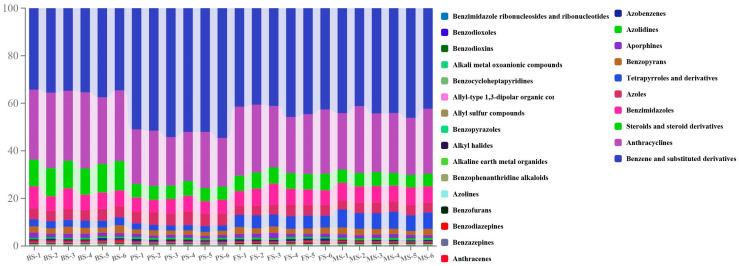
Stacked column chart of metabolite classes in *Ganoderma lucidum* at different growth stages.

To gain deeper insights into the variations occurring during the different developmental stages of *G. lucidum*, we performed metabolomics analysis using LC-MS/MS and determined the metabolite content. The four samples of BS, PS, FS, and MS were processed for comparison. The result of metabolomic analysis has yielded a comprehensive catalog of 2043 metabolites(Figure 2), including, but not limited to, 387 glycerolipids, 326 benzene and substituted derivatives, 283 organooxygen compounds, 280 carboxylic acids and derivatives, 207 prenol lipids, 143 fatty acyls, 132 glycerophospholipids, 105 steroids and steroid derivatives, 94 purine nucleotides, 86 pyrimidine nucleotides, 78 flavonoids, 43 pyridines and derivatives, 42 organic phosphoric acids and derivatives, 40 tannins, 39 azoles, and 38 quinolines and derivatives (Supplementary Table S1).

**Figure 3:**
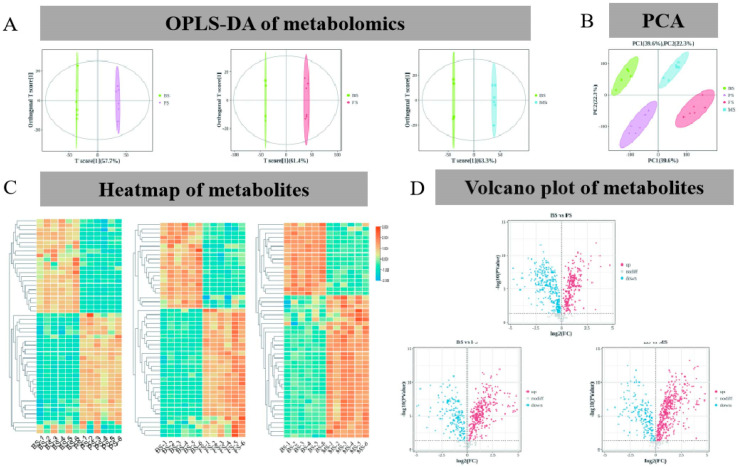
Multivariate analysis of the metabolome and proteome of *G. lucidum* at different growth periods. (**A**) Metabolome PCA; (**B**) Metabolome OPLS-DA; (**C**) Metabolite heatmap (TOP50); (**D**) Metabolome volcano map.

PCA was employed to characterize the metabolomic data of various samples by considering the levels of the components. The replicates of every sample were grouped together and distinguished from the rest of the samples. The initial two principal components (PCs) explained 61.9% of the variability, with PC1 representing 39.6% and PC2 representing 22.3%. It is noteworthy that PC1 exhibited a steady rise with the progression of the growth phase (Figure 3A), indicating dynamic changes in metabolites during the growth period. Additionally, the results of OPLS-DA showed that the periods were segregated; score plots explained 57.7% (BS–PS), 61.4% (BS–FS), and 63.3% (BS–MS) of the variation in the two components (Figure 3B).

The normalized metabolites were quantified and visualized to compare differences among samples, as shown in Figure S2. Significant differences in metabolite profiles were observed as the growth period progressed, with this trend becoming increasingly apparent over time. Upon classifying the content of *G. lucidum*, it was noted that certain metabolites were more prevalent during specific stages. Steroids and steroid derivatives, cinnamic acid and derivatives, glycerol lipids, glycerophospholipids, pyrimidines, and nucleoside metabolites were found to be more abundant in BS. Organonitrogen compounds, imidazopyridines, benzene and substituted derivatives, and azole metabolites were higher during PS. Nucleotide metabolites were more prevalent during FS, while purine nucleotides, isoflavonoids, thiazines, pteridines and derivatives, and phenanthrene and derivatives were at their peak during FS. Tetrahydropyrrole and tetrahydropyrrole derivatives, organic oxides, carboxylic acids and derivatives, fatty acyl groups, purine nucleotides, glycerophospholipids, and pyrimidine nucleotides were most abundant during MS.

To identify significant differential metabolites, we applied a set of criteria: VIP values exceeding 1.0 and |log2(Fold change)| ≥ 1 (*p* < 0.05). These criteria allowed us to obtain significantly differential metabolites from the paired-comparison groups, including BS, PS, FS, and MS. As shown in Figure 3C, the number of significantly different metabolites (DEMs) increased throughout the developmental stages of *G. lucidum*. With 400 upregulated and 194 downregulated metabolites, the BS/FS group had the greatest number of metabolites. Furthermore, metabolites were found to be 234 upregulated and 304 downregulated in the BS/PS group and 325 upregulated and 189 downregulated in the BS/MS group (Figure 3D and Table 2).

Furthermore, we screened the top 50 differentially expressed metabolites based on their abundance and *p*-values. The results revealed significant changes in the metabolic composition of *G. lucidum* during the different developmental periods. In the color-coded representation, blue indicates lower metabolite concentrations and red indicates enhanced metabolite levels. For instance, the abundance of mizolastine, geranlin, teritramine, chicoric acid, and chrysoeriol increased significantly between BS and PS. On the other hand, the abundance of erythronic acid, eriocitrin, pseudopurpurin, isoimide, and suberic acid decreased significantly. Between BS and MS, trehalose, avenanthramide C, xenognosin B, and perfluorohexane sulfonic acid increased significantly, whereas filiforminol, kynurenic acid, cortisol, remoxipride, and fenoxanil levels decreased significantly. When comparing BS and FS, nitisinone, perillic acid, temazepam, atantolactone, and aurothioglucose levels decreased significantly, whereas xanthosine, succinic acid, sytyene, and succinyladenosine levels increased (Figure 3C,D).

### 2.3. Changes in Triterpenoid Content of G. lucidum during Different Growth Periods

**Figure 4:**
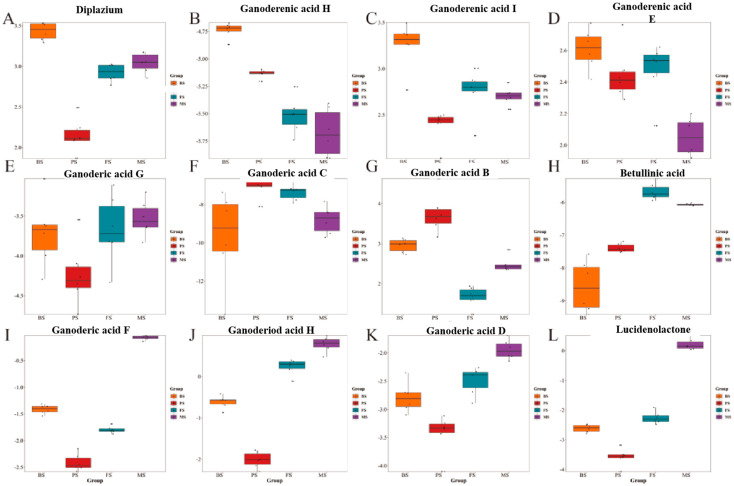
Box plot of triterpenoid content: The box plots display the distribution of triterpenoid content in *G. lucidum* at different growth stages. (**A**) Diplazium, (**B**) Ganoderenic acid H, (**C**) Ganoderenic acid I, (**D**) Ganoderenic acid E, (**E**) Ganoderic acid G, (**F**) Ganoderic acid C, (**G**) Ganoderic acid B, (**H**) Betullinic acid, (**I**) Ganoderic acid F, (**J**) Ganoderiod acid H, (**K**) Ganoderic acid D, (**L**) Lucidenolactone.

*Ganoderma lucidum* triterpenoids possess a wide array of medicinal properties, including anti-inflammatory, antioxidant, immunomodulatory, and anticancer effects, which are crucial for disease prevention and treatment. Their ability to inhibit cancer, strengthen the immune system, and provide antioxidative properties highlights their importance as a natural compound with significant health benefits. In this study, a total of 12 triterpenoids were identified, with five of them present in high relative abundance in BS, such as Diplazium and Ganoderenic acids E, H, and I, while Ganoderenic acids B and C were abundant in PS species. Additionally, Ganoderenic acids B and C were higher in PS species, with only Euscaphic acid being more abundant in FS, and the rest showing higher levels in MS (Figure 4).

### 2.4. Changes in Steroid Content of G. lucidum during Different Growth Periods

**Figure 5:**
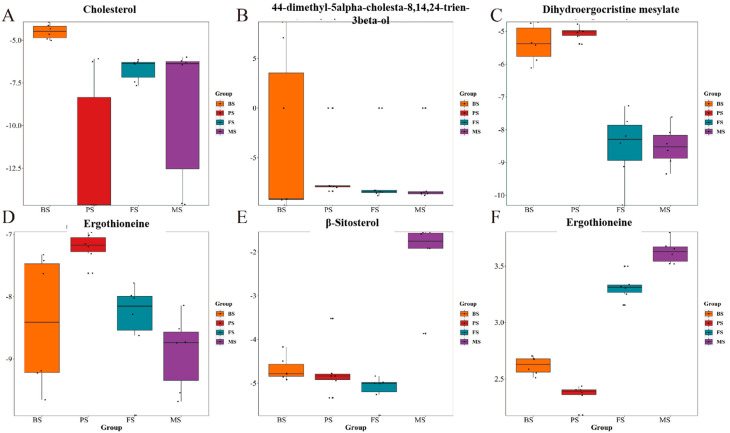
Box plot of steroid content: The box plots illustrate the distribution of steroid content in *G. lucidum* at different growth stages. (**A**) Cholesterol, (**B**) 44-dimethyl-5alpha-cholesta-8,14,24-trien-3beta-ol, (**C**) Dihydroergocristine mesylate, (**D**) Ergothioneine, (**E**) β-Sitosterol, (**F**) Ergothioneine.

Steroids, a crucial secondary metabolite found in *G. lucidum*, exhibit diverse medical applications. They showcase a plethora of pharmacological benefits, including anti-inflammatory, antioxidant, immunomodulatory, and anti-cancer properties, pivotal for disease prevention and treatment. The anti-cancer effects, immune system fortification, and antioxidant capabilities of steroids underscore their significance as a natural compound with promising health advantages. In this study, a total of eight steroids were identified. Among them, cholesterol and 4,4-dimethyl-5alpha-cholesta-8,14,24-trien-3beta-ol exhibited high relative abundance in BS. Dihydroergocristine mesylate showed a high relative abundance, while dihydro-alpha-ergocryptine mesylate had a high relative abundance in PS. Ergothioneine and cabergoline were predominantly found in MS (Figure 5).

### 2.5. Proteomic Results of G. lucidum at Different Growth Periods

**Figure 6:**
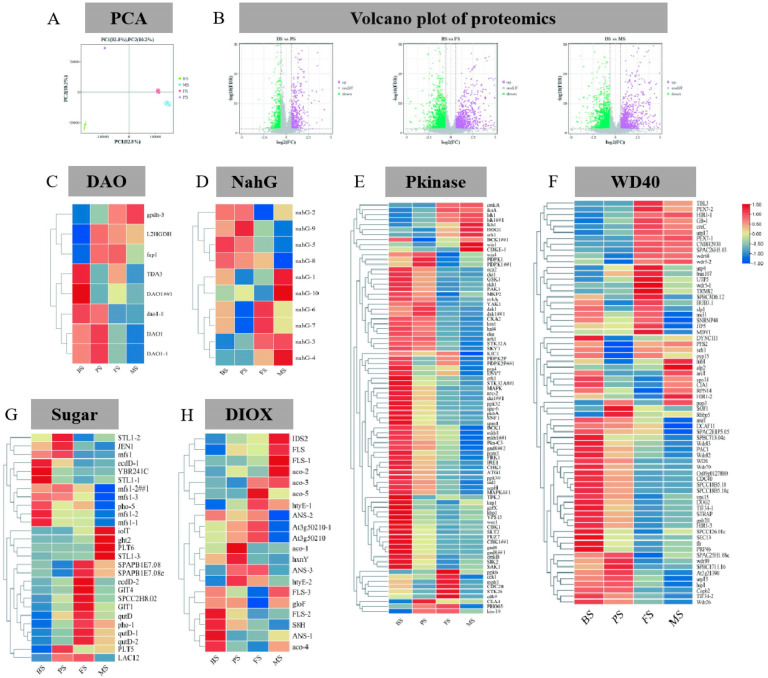
Multivariate analysis of the proteome of *G. lucidum* at different growth periods. (**A**) Proteome PCA; (**B**) Proteome volcano map; (**C**) DA0; (**D**) NahG; (**E**) Pkinase; (**F**) WD40; (**G**) Sugar; (**H**) DIOX.

A DIA detection approach was used to identify and quantify 6647 proteins. The PCA results effectively distinguished the proteomic profiles of the different samples, as shown in Figure 6A. The first two principal components explained 93.0% of the total variance. Furthermore, the analysis focused on six protein families (DAO, NaGh, Pkinase, WD40, sugar, DIOX) associated with *G. lucidum* growth and development. Among these families, DAO-1, DAO1-1, and TDA3 exhibited high expression levels in the BS, as shown in Figure 6B. Similarly, NahG showed a higher expression in the BS (Figure 6C). Pkinases regulate the function and activity of target proteins through phosphate group transfer, representing the active state of an organism. Most of the Pkinases were more active in the BS, as illustrated in Figure 6D. WD40 proteins, which are involved in various cellular processes such as signal transduction, protein degradation, and chromatin remodeling, play crucial roles in gene expression regulation, cell cycle progression, and development. These proteins were highly expressed in both the BS and FS (Figure 6F). Sugar proteins play a vital role in the regulation of sugar metabolism and energy homeostasis within cells. It exhibited higher activity primarily in the FS (Figure 6G). DIOX, a significant oxidoreductase involved in alkaloid synthesis, showed varied expression patterns in different samples. FLS-2, S8H, and ANS-1 were highly expressed in BS, aco-1 and hxnY were highly expressed in PS, aco-5 was highly expressed in FS, and IDS and FLS-1 were highly expressed in MS (Figure 6H).

### 2.6. Changes in Proteins Related to Triterpene and Steroid Synthesis Content of G. lucidum during Different Growth Periods

**Figure 7:**
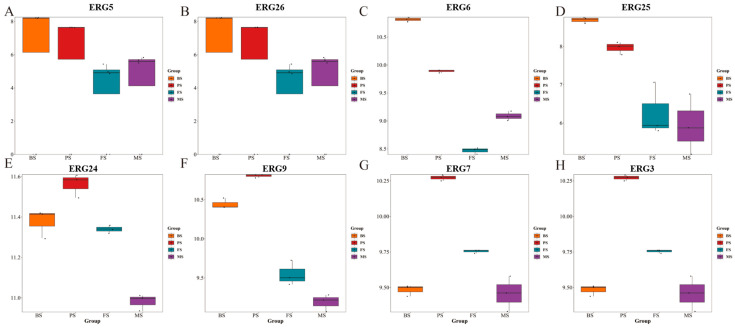
Box plot of ERG protein content: The box plots show the distribution of ERG protein family content in *G. lucidum* at different growth stages. (**A**) ERG5, (**B**) ERG26, (**C**) ERG6, (**D**) ERG25, (**E**) ERG24, (**F**) ERG9, (**G**) ERG7, (**H**) ERG3.

The biosynthesis pathway of triterpenoids and steroids in *G. lucidum* has been a focal point for researchers. Some key proteins play vital roles in elucidating this synthesis mechanism. Among these proteins, HMGR and HMGS have been extensively investigated. This study revealed that the ERG protein family also holds significant importance in the synthesis of triterpenoids in *G. lucidum*. A total of eight ERG family proteins were identified, with the majority displaying a decreasing expression trend as *G. lucidum* matures. Furthermore, the AREG family of proteins, specifically AREG1 and AREG2, exhibited an escalating trend in expression levels as *G. lucidum* progressed in growth (Figure 7).

### 2.7. WGCNA and Color Module Identification of Metabolomic at Different Growth Periods

**Figure 8:**
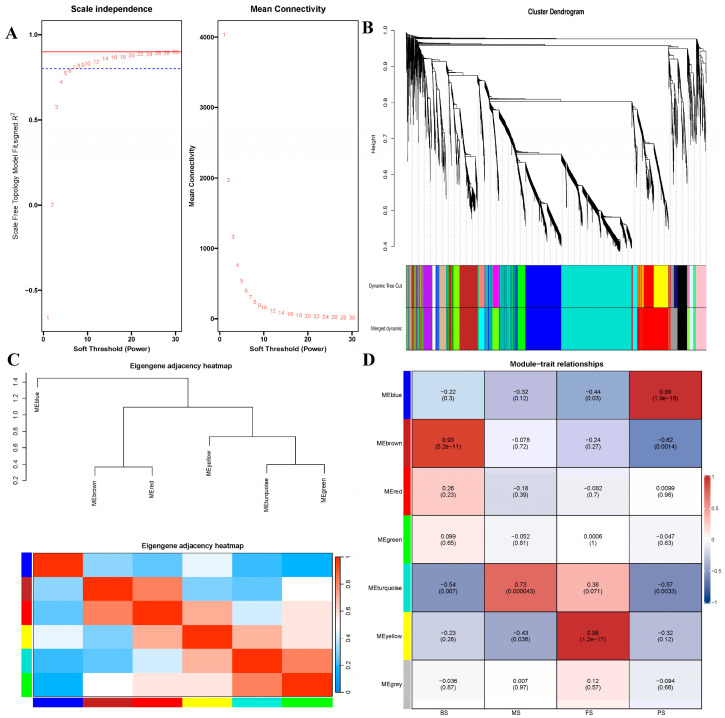
Metabolic WGCNA. (**A**) Analysis of network topology for various soft-thresholding powers. The left panel shows the scale-free fit index (*y*-axis) as a function of the soft-thresholding power (*x*-axis). The right panel displays the mean connectivity (degree, *y*-axis) as a function of the soft-thresholding power (*x*-axis); (**B**) Clustering dendrogram of proteomics, with dissimilarity based on topological overlap, together with assigned module colors; (**C**) Clustering dendrogram of samples with trait heatmap; (**D**) Module-trait associations: Each row corresponds to a module eigengene and each column to a trait. Each cell contains the corresponding correlation and *p*-value. The table is color-coded by correlation according to the color legend.

The selected metabolomics were clustered by employing a dissimilarity measure based on a topological overlap matrix (TOM). This clustering method utilized a dynamic tree-cutting algorithm to divide the tree into seven modules, with each module distinguished by a unique color (Figure 8A,B). Every module aligned with a distinct metabolomic cluster, as shown in the heat map with overlapping topology. In this map, the color red indicates a positive correlation, while blue signifies a strong negative correlation (Figure 8C). We outlined the co-expression patterns of distinctive metabolites and computed the correlation between each unique metabolome and the corresponding growth cycle stage: BS, PS, FS, and MS. This analysis involved assessing the metabolites within each module, which are indicative of the primary metabolite expressions within the module, and determining the Spearman’s correlation coefficient for each growth cycle stage. The results of the modulotrait analysis indicated the following correlations in the metabolomic co-expression diagram: metabolites from the brown module correlated positively with BS (cor = 0.93, *p* < 0.001), metabolites from the blue module correlated positively with PS (cor = 0.99, *p* < 0.001), metabolites from the yellow module correlated positively with FS (cor = 0.98, *p* < 0.001), and metabolites from the turquoise module correlated positively with MS (cor = 0.73, *p* < 0.001) (Figure 8D). 

### 2.8. WGCNA and Color Module Identification of Proteins at Various Growth Periods

Based on an R^2^ value of 0.88, a soft threshold of β = 9 was applied to the lncRNA expression matrix to construct a scale-free network with a scale-free topological fit index greater than 0.85 (Figure S3A,B). Furthermore, a topological overlapping heat map was generated, depicting the correlation between the characteristic proteins and color coding (Figure S3C). Upon conducting the modulotrait analysis of the proteomic co-expression diagram, it was revealed that the turquoise module in proteomics (correlation coefficient = 0.79, *p* < 0.001) exhibited a positive correlation with the BS. Similarly, the green module in proteomics (correlation coefficient = 0.97, *p* < 0.001) exhibited a positive correlation with the PS. Additionally, the yellow and blue proteomics modules (correlation coefficient = 0.97, *p* < 0.001) were positively correlated with FS. Finally, the yellow module in proteomics (correlation coefficient = 0.76, *p* < 0.001) was positively correlated with MS (Figure S3D).

### 2.9. Integrated Analysis of the Metabolome and Proteome

**Figure 9:**
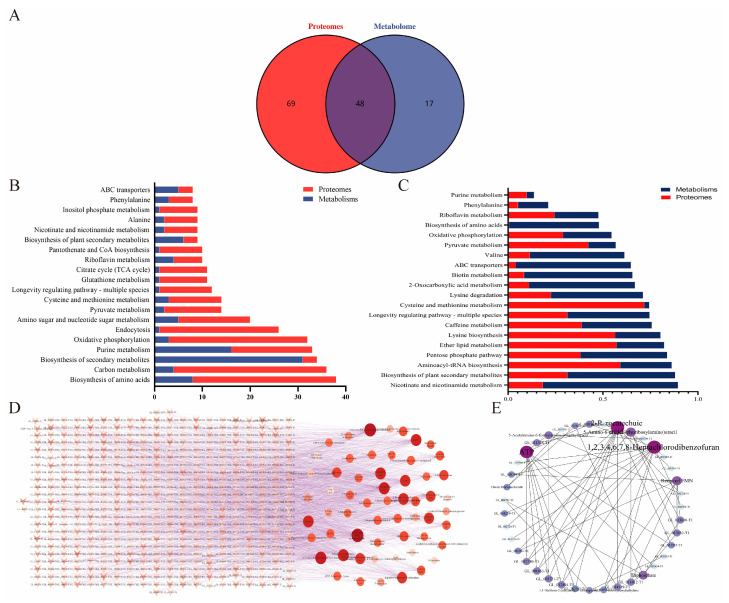
(**A**) Venn diagram of the KEGG pathways in which differentially accumulated metabolites and abundant proteins were involved; (**B**) The top 20 KEGG pathways contain the most metabolites and proteins; (**C**) The top 20 KEGG pathways enriched with metabolites and proteins (ranked by *p*-value); (**D**) Network analysis constructed using all differentially accumulated metabolites and abundant proteins; (**E**) Network analysis between metabolites and proteins in key pathways.

Color modules with high expression levels of metabolites and proteins in the FS were identified based on the WGCNA results, and KEGG pathways were selected as the carriers. After that, an integrative analysis was done. A Venn diagram was used to display the KEGG pathway numbers related to the proteome and metabolome (Figure 9A). The number of KEGG pathways linked to the abundant proteins and differently accumulating metabolites found by the two omics techniques is shown by the cross-sections in the circles (Supplementary Table S2). The proteome and metabolome were shown to involve 48 common pathways, which comprised purine metabolism, oxidative phosphorylation, amino acid biosynthesis, carbon metabolism, and the formation of secondary metabolites. The top 20 KEGG pathways with the most proteins and metabolites were identified by examining the 48 common pathways (Figure 9B). The top 20 KEGG pathways ranked based on *p*-value are shown in Figure 9C. The higher the bar from bottom to top, the more active the biological pathway in the measurement sample. These proteins and metabolites appear to be mostly engaged in primary and secondary metabolisms, according to these integrated omics investigations. It was recently suggested that secondary metabolism plays a critical role in the growth and development of fungi. The primary routes for riboflavin metabolism, phenylalanine metabolism, purine metabolism, and secondary metabolism were represented by the common pathways in Figure 9B,C. Using Cytoscape, a network analysis of abundant proteins, KEGG pathways, and differentially accumulated metabolites was carried out in order to gain a better understanding of the relationship between metabolomics and proteomics.

The EFAAH, bos1, Abcb6, pzh1, SNU13, tartaric acid, ceramide, ATP, dATP, avermectin B1b monosaccharide, and biochanin A had the lowest *p*-values and were directly involved in the purine metabolism and ribosome biogenesis pathways, as shown in Figure 9D. Since a protein may interact with several metabolites when it is a co-pathway partner and a metabolite can integrate with numerous proteins, we further integrated these important proteins and metabolites with associated pathways in a single network. The major pathways included thiamine metabolism, folate biosynthesis, ABC transporters, phenylalanine, tyrosine, and tryptophan biosynthesis, as well as riboflavin metabolism pathways (Figure 9E).

**Figure 10:**
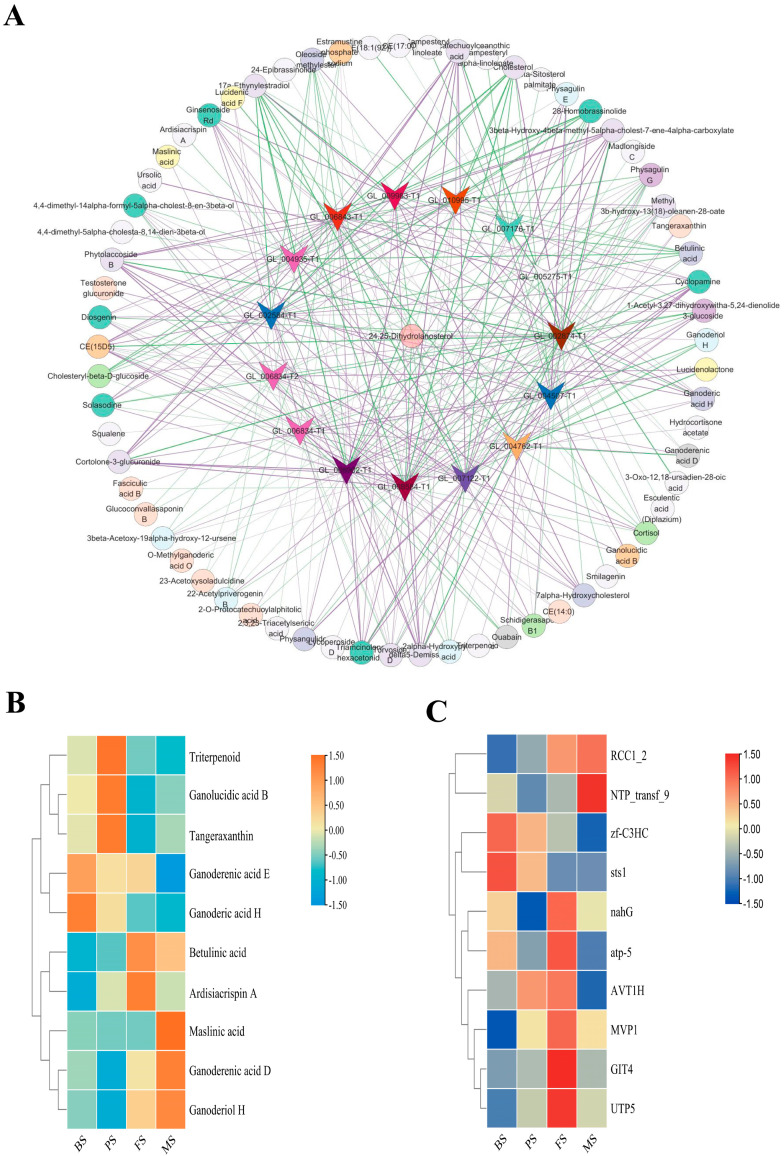
(**A**) Network analysis constructed using triterpenoid metabolites and proteins; (**B**) Hub proteins heatmap (TOP 10); (**C**) Hub metabolites heatmap (TOP 10).

We conducted correlation analysis on triterpenoid metabolites and related proteins using Cytoscape and the results showed that a total of 667 expression metabolite associations were identified across multiple omics networks, involving 66 metabolites and 15 proteins (Figure 10A), and the research results showed that SPCC1672.09, ERG3, Sqle, sts1, ERG9, erg5, and physagulin were strongly correlated. Next, the top ten metabolites and proteins were selected according to their degrees of connectivity to create a cluster heat map (Figure 10B,C). The results showed that six proteins were highly expressed in FS and the levels of Ganoderenic acid E and Ganoderic acid H were the highest in BS. Triterpenoid, ganolucidic acid B, and tangeraxanthin had the highest PS content. There was a high content in FS of betulinic acid and ardisiacrispin A. Maslinic acid, ganoderiol acid H, and Ganoderenic acid D had a higher content in MS.

## 3. Discussion

Medicinal and edible mushrooms have played a significant role in human diets and traditional medicine for a considerable period. *G. lucidum*, a species of both culinary and therapeutic importance, has been documented in ancient Chinese medicinal literature, such as the Shennong Materia Medica Classic and Compendium of Materia Medica, and mostly appears in the text of Liuzhi under the name Red Chi, to which *G. lucidum* belongs [5,6]. Functional foods such as *G. lucidum* tea, with *G. lucidum* as the principal ingredient, have gained significant popularity among the Chinese population. However, the cultivation of *G. lucidum* is challenging due to the lack of understanding of its metabolites and their underlying mechanisms during distinct growth and developmental stages, as well as the absence of a standardized quality control system for fruiting bodies.

In the present study, a comprehensive analysis identified 2043 metabolites, representing a wide range of chemical compounds. These metabolites encompass various categories, including glycerides, benzenes and substituted derivatives, organooxides, carboxylic acids and derivatives, proalcohol lipids, fatty acyl groups, glycerol phospholipids, steroids and steroid derivatives, purine nucleotides, pyrimidine nucleotides, flavonoids, pyridines and derivatives, organic phosphoric acids and derivatives, tannins, azole compounds, and quinoline and its derivatives, among others. This analysis provided valuable insights into the diverse compositions of the metabolites present in the studied samples.

Notably, *G. lucidum* is particularly rich in fatty acids, glycerides, and prenol lipids, which account for 8–15% of its dry weight. Metabolomic analysis revealed that these compounds were the most abundant in the fruiting bodies of *G. lucidum*. These compounds are essential for human nutrition, as they contribute to increased physical activity and metabolic rates. Additionally, they play crucial roles in various cellular activities via their receptors, making them essential nutrients for the growth and development of *G. lucidum*. The energy derived from these compounds supports the synthesis of cellular components and the maintenance of physiological function growth. Moreover, it serves as a source of energy and carbon, promoting the growth and development of *G. lucidum* and facilitating the production of secondary metabolites, such as triterpenoids and polysaccharides, through metabolic pathways.

**Figure 11:**
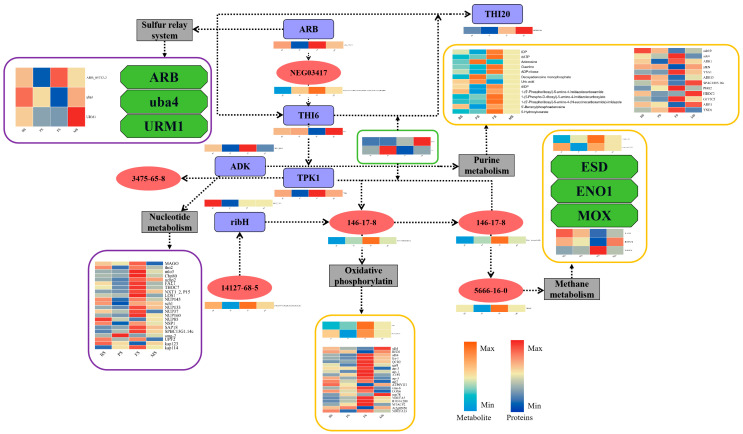
Integrated metabolic network diagram. The heatmap was plotted using log2-transformed values from the metabolomic and proteomic dataset, and the cells in the heatmap from left to right represent BS, PS, FS, and MS samples, respectively. 3475-65-8: Thiamin triphosphate; 1427-68-5: GTP; 146-17-8: Riboflavin; 5666-16-0: Reduced FMN; 3475-65-8: Thiamin triphosphate; NEG03417: 2-[(2R,5Z)-2-Carboxy-4-methylthiazol-5(2H)-ylidene]ethyl phosphate; ribH: 6,7-dimethyl-8-ribityllumazine synthase [EC:2.5.1.78]; ADK: adenylate kinase; TPK1: hiamine pyrophosphokinase [EC:2.7.6.2]; ARB: ARB_05732-2; THI20: hiamine-phosphate diphosphorylase/hydroxyethylthiazolekinas.

By using comparative metabolomics and proteomics analysis, we were able to determine and describe how *G. lucidum* modifies important metabolic pathways differently. These pathways include phenylalanine, tyrosine, tryptophan biosynthesis, riboflavin metabolism, ABC transporters, thiamine metabolism, methane metabolism, and folate biosynthesis. We describe in the next sections the functional groups and corresponding metabolic processes of these differently accumulated metabolites and abundant proteins, as illustrated in Figure 11. These results offer important new understandings of the complex regulatory processes that underpin *G. lucidum* growth and development. By understanding the specific metabolic pathways and proteins involved at different stages of *G. lucidum* growth, we acquire a better understanding of its overall metabolic profile and biosynthetic mechanisms. This knowledge contributes to improving the quality control of *G. lucidum* fruiting bodies and holds potential for the development of new functional foods and medicinal products derived from this valuable fungus.

### 3.1. Impact of G. lucidum at Different Growth Periods on Phenylalanine, Tyrosine, and Tryptophan Biosynthesis

Fungi gather a variety of aromatic amino acids throughout growth and development, including tyrosine and phenylalanine, which are needed as building blocks for primary and secondary metabolites in biosynthesis. According to a prior study, throughout the development period, phenylalanine and tyrosine levels dramatically rose [18]. Tyrosine and phenylalanine levels were likewise markedly elevated in this investigation, with comparable outcomes. Additionally, markedly upregulated in FS were the expressions of histidinol-phosphate aminotransferase (his3), aromatic amino acid aminotransferase I/2-aminoadipate transaminase (AR08), and D-Erythrose 4-phosphate. There are two possible reasons for the increase in phenylalanine and tyrosine levels. First, his3, AR08, and phenylpyruvate are the last steps in the biosynthesis of phenylalanine and tyrosine and play a crucial role in this process. Therefore, increased levels of phenylalanine and tyrosine may be related to increased expression of his3a and AR08. Second, phenylalanine and tyrosine can be used as precursors for lignin synthesis in the phenylpropanoid biosynthesis pathway. Therefore, an increase in lignin synthetase abundance may lead to an increase in phenylalanine and tyrosine content in *G. lucidum*.

### 3.2. Impact of G. lucidum at Different Growth Periods on Energy Production

During the growth of *G. lucidum*, physiological activities such as morphological development and spore formation require energy. Macromolecular hydrolysates are also used for energy production.

Riboflavin, also known as vitamin B2, plays a crucial role in energy metabolism and acts as an antioxidant [19], immune function regulator, and nervous system and skin health regulator. Flavin mononucleotide (FMN) was significantly upregulated during the growth of *G. lucidum*. FMN is a coenzyme [19]. Flavin adenine dinucleotide (FAD) and riboflavin (vitamin B2) are crucial components of biological oxidation processes, such as respiration. They serve as coenzymes, specifically belonging to the flavinase group, and participate in electron transfer from the substrate to the electron acceptor upon binding to the apo-enzyme. Riboflavin is also involved in the metabolism of glucose, fat, and proteins, thereby facilitating the conversion of food into energy. Furthermore, FMN, a riboflavin derivative, can directly enter the oxidative phosphorylation pathway, thereby providing energy for the growth and development of *G. lucidum*. In addition to its role in energy metabolism, FAD synthetase catalyzes FMN adenylation to form FAD. This enzyme was significantly upregulated and played a vital role in oxidative phosphorylation and purine metabolism. Specifically, it catalyzes the condensation of 5-amino-6-(D-ribitylamino) uracil with 3,4-dihydr-oxy-2-butanone 4-phosphate, leading to the formation of 6,7-dimethyl-8-ribityllumazine. Overall, these coenzymes, including FMN and FAD, are essential for various physiological processes, including electron transfer, energy production, and metabolic pathways. Their upregulation during the growth of *G. lucidum* indicates their importance in facilitating the development of the organism and energy production.

### 3.3. Impact of G. lucidum at Different Growth Periods on ABC Transporters

ABC transporters are essential for transporting complex organic matter across concentration gradients. Their ability to transport such compounds renders them well-suited for providing the building blocks required for the development of specialized cells and organisms. ABC transporters play crucial roles in *G. lucidum* growth and development. They are involved in various processes, including mycelium formation, spore germination, organogenesis, and secondary growth. These transporters are directly powered by ATP, which enables the transport of complex organisms across concentration gradients [20,21].

In our study, a significant upregulation of 4-Trimethylaminobutyric acid (TMABA) was observed during ABC transport. TMABA is a positively charged quaternary ammonium salt that acts as a substrate for translocation through ABC transporter proteins. It has been postulated that TMABA facilitates substrate transport by interacting with the binding sites of ABC transporter proteins and inducing conformational changes in these proteins. Furthermore, we observed a significant downregulation of L-Aspartate, which undergoes extracellular transport by binding to substrate-binding proteins. This process involves ATP hydrolysis and conformational changes. In addition, L-Aspartate also plays a role in promoting the growth and development of *G. lucidum* through its involvement in purine metabolism.

Overall, the high expression of ABC transporters in *G. lucidum* may be attributed to the requirement of metal elements for enzyme binding. This interaction helps regulate the activity and structure of the enzymes, thereby maintaining the morphological features, growth, and development of *G. lucidum* during its growth process. These processes contribute to the promotion of *G. lucidum*’s growth and development and the maintenance of its phenotypic features.

### 3.4. Variations in Triterpenes and Their Synthesis during the Development of G. lucidum

Triterpenes are a specific group of terpenes that can be found in various medicinal plants in nature. When it comes to *G. lucidum*, the triterpenes isolated from this species are primarily bioactive constituents known as ganoderic acids. These triterpenes exhibit a diverse range of biological activities and are characterized by a highly oxygenated lanostane chemical structure [22,23]. A total of 65 triterpenoids were identified using metabolomics. The number of triterpenoid DEMs decreased progressively with the extension of the growth period, and the highest number of species was observed in the BS (Figure S4). Among these species, ganoderic acid I, ganoderic acid H, and ganoderic acid E exhibited the highest levels during BS, whereas ganoderic acid C and ganolucidic acid B were highest during PS. Eburicoic acid showed higher levels during the FS, and ganoderiol H and ganoderic acid D were higher during the MS. In conclusion, triterpenoids are higher in BS and the types and contents of the active ingredients in *G. lucidum* undergo constant changes throughout its growth stages. These changes are closely linked to the metabolic transformation of the fungus and its surrounding environment during each growth stage. Elucidation of the biosynthetic pathways of sterols and triterpenes in *G. lucidum* is important for further research on the medicinal value of *G. lucidum*. Currently, the biosynthetic pathway of triterpenes in Ganoderma consists of more than 20 consecutive enzymatic reactions. Meanwhile, acetyl coenzyme A can be used as a prerequisite for the synthesis of ganoderic acid through the MVA pathway. The MVA pathway is the primary mechanism for triterpene synthesis. Hydroxymethylglutaryl-CoA reductase (HMGR) is the first rate-limiting enzyme in the MVA pathway [24]. HMGR (hydroxymethylglutaryl-CoA reductase) promotes the synthesis of chemo mevalonate, a specific precursor for all isoprenoid compounds present in plants. HMGR plays an important role in the MVA pathway by promoting triterpenes accumulation in *G. lucidum* and directing the synthesis of several triterpenoids. High expression of this protein during the BS promotes the synthesis of triterpenoid compounds with different conformations, such as ganoderic acid I, ganoderic acid H, and others. Cytochrome P450 monooxygenase is a widely distributed oxidoreductase in organisms that catalyzes a variety of reactions such as hydroxylation, epoxidation, dealkylation, sulfation, deamidation, desulfurization, dehalogenation, and nitroreduction. After the synthesis of lanosterol, the carbon skeleton undergoes a series of modification reactions to generate structurally different triterpenoids, and CYP450 is involved in the carbon skeleton modification reactions of triterpenoids in plants. In plants, CYP450 is involved in the carbon skeleton modification reactions of triterpenoids, which have not been reported in *G. lucidum*, and this result was better verified by the high expression of P450-4 and P450-2 in BS. As the most important active substance in *G. lucidum*, ganoderic acid has good pharmacological activity; however, the mechanism of phase synthesis is still unclear and needs to be further investigated.

The material studied in this paper was the cultivated *G. lucidum* fruiting bodies of the wild strain from Luoshan, Shandong, China. Whether other strains of *G. lucidum* from other locations follow the same pattern will be the subject of further comparative studies.

## 4. Materials and Methods

### 4.1. Materials and Chemicals

The samples used in this study were obtained from the Institute of Xixun Fungal Research. These samples were domesticated and cultivated from wild strains of *G. lucidum* collected in Luoshan, Yantai City, Shandong Province, China. The internal transcribed spacer (ITS) sequence of the identified species was obtained and documented (Figures S1 and S2). The specimen has been deposited in the Fungarium of Jilin Agricultural University Changchun, China (reference number HMJAU70062).

As seen in Figure 1A, the fruiting bodies of *G. lucidum* were collected at four different developmental phases, each of which was distinguished by particular morphological alterations. In stage 1, the stipe measured approximately 3 cm in length, lacked a cap, and displayed a hemispherical tip. Progressing to stage 2, the cap tip exhibits a white-to-yellow coloration. During stage 3, the cap assumed a semicircular or renal shape and displayed a yellow hue. Finally, in stage 4, the fruiting body developed branches, and its color gradually darkened, transitioning to a brownish shade.

Twelve experimental samples were collected at each time point, specifically from the mushroom caps. Following collection, the samples were immediately preserved in liquid nitrogen and subsequently stored in a refrigerator at −80 °C until metabolomics and proteomics analyses. The samples were stored at the Key Laboratory of Medicinal Resources Development and Utilization, Jilin Agricultural University.

The study utilized high-quality chemicals for analysis. Merck (Darmstadt, Germany) provided acetonitrile, methanol, and formic acid. Tedia (Osaka, Japan) supplied acetic acid and methyl alcohol. Deionized water underwent purification through a Milli-Q water purification system by Millipore (Billerica, MA, USA).

### 4.2. Sample Extraction

#### 4.2.1. Metabolite Extraction

After being swiftly frozen in liquid nitrogen, the mushroom samples were ground into a fine powder with a mortar and pestle. To extract metabolites, a homogenization solution comprising 1000 μL of methanol/acetonitrile/H_2_O (2:2:1, *v*/*v*/*v*) was added. Subsequently, the mixture underwent centrifugation at 14,000× *g* and 4 °C for 15 min, followed by vacuum drying of the supernatant. For the liquid chromatography-mass spectrometry (LC-MS) analysis, the dried samples were reconstituted in a solvent composed of 100 μL acetonitrile/water.

#### 4.2.2. Protein Extraction

With slight adjustments, the previously outlined methodology was followed for the extraction and digestion of proteins [25]. Frozen tissue samples (200 mg) were vortexed with 500 μL of cold extraction buffer (100 mM Tris-HCl pH 8.5, 5 mM ethylenediaminetetraacetic acid, 100 mM potassium chloride, 1% (*w*/*v*) dithiothreitol, 1 mM phenylmethylsulfonyl fluoride, and 30% (*w*/*v*) sucrose). Afterward, 500 μL of phenol solution in triple equilibrium was included, and the blend was homogenized at 4 °C for 10 min, then centrifuged at 10,000× *g* for 10 min at 4 °C. The phenol phase underwent a second extraction with 500 μL of extraction buffer and was subsequently incubated overnight at −20 °C. Protein precipitation was achieved by adding 1 mL of 0.1 M ammonium acetate methanol solution, followed by centrifugation at 17,000× *g* for 30 min at 4 °C to remove the supernatant. The resulting pellet was washed with 0.2% (*w*/*v*) dithiothreitol (DTT) in cold acetone, incubated for 1 hat -20 °C, and centrifuged again. The pellet was air-dried, resuspended in 100 μL lysis buffer (8 M urea, 5 mM DTT, and 30 mM Tris), and vortexed to dissolve the proteins. Protein quantification was performed using a Bio-Rad (Hercules, CA, USA) Protein Assay with bovine serum albumin (BSA) as a standard. Later, 20 μg of protein was diluted with DTT and incubated with iodoacetamide in the dark. The sample was diluted with 0.15 M ammonium bicarbonate, and trypsin was added for protein digestion at 37 °C. The obtained peptides were acidified with a trifluoroacetic acid solution, purified using a Pierce C18 spin column, and the solvent was evaporated using a Speed-Vac evaporator. The final pellet was dissolved in 0.1 M ammonium formate.

In order to guarantee the quality of the data for metabolic analyses, QC samples were generated by merging segments from each of the samples. These QC samples represented the entire set of samples and were used for data normalization. Moreover, the QC samples were subjected to the same preparation and analysis procedures as those utilized for each batch of experimental samples.

### 4.3. Determination of Nutrient Composition and Micro- and Macro-Elements

In this study, several parameters were analyzed, including triterpenes, sterols, crude protein, total phenols, crude polysaccharides, and total flavonoids, as well as the elemental compositions of Cu, Fe, K, Mg, Na, Zn, and Se [26].

The phenol-sulfuric acid method was employed to quantify the total polysaccharides, while the aluminum nitrate colorimetric method and the Folin-Ciocalteu colorimetric method were used to determine the total flavonoids and total polyphenols, respectively. Additionally, the quantification of total triterpenoids followed the vanillin-perchloric acid method, in accordance with guidelines T/AHFIA 004-2018, SN/T 4260-2015, SN/T 4592-2016, T/AHFIA 005-2018, and NY/T 3676-2020. The sulfate-phosphate-ferric method was utilized for the determination of total sterols, and the BCA kit (Pierce, Rockford, IL, USA) was employed for the analysis of crude proteins and determination of amino acids, following the manufacturer’s instructions, with reference to GB5009.124-2016. For elemental determination, 5 mL of nitric acid was added to 0.2 g of the sample and then subjected to microwave digestion, involving a stepwise increase in temperature up to 190 °C for 15 min. The solution was diluted to 50 mL after cooling and analyzed for elemental content using an ICP-MS unit (iCAPQ, Thermo Scientific, San Jose, CA, USA) with specific operational parameters. Detailed procedures can be found in  Supplementary File S1.

### 4.4. Metabolomic Analysis

Liquid chromatography-mass spectrometry (LC-MS) analysis was conducted in this study using a UHPLC-Q Exactive HF-X system. The raw data files obtained from LC-MS were processed using Compound Discoverer 3.1 (CD3.1, Thermo Fisher, Waltham, MA, USA) software. This software was utilized for peak alignment, peak picking, and quantification of individual metabolites. Detailed methodologies can be found in Supplementary File S2.

### 4.5. HPLC-MS/MS Gel-Free Proteomic Analysis

Extraction of samples was performed on 0.5 μg of digested material using a Dionex Ultimate 3000 Ultra High-Performance Liquid Chromatography system (Thermo Scientific, Rockford, IL, USA). The system was equipped with a Thermo Scientific C18 PepMap RSLC column (2 μm, 50 μm–15 cm). The mobile phase consisted of solvent A (0.08% (*v*/*v*) formic acid dissolved in water (Sigma-Aldrich, St. Louis, MO, USA) and solvent B (0.08% (*v*/*v*) formic acid dissolved in 80% (*v*/*v*) acetonitrile, Sigma-Aldrich, St. Louis, MO, USA). Elution was carried out using a linear gradient at a flow rate of 300 μL/min. The gradient started with 0–4% buffer B for 3 min, followed by 4–10% buffer B for 12 min, 10–35% buffer B for 20 min, 35–65% buffer B for 5 min, and 65–95% buffer B for 1 min. Subsequently, the system was maintained at 95% buffer B for 10 min, followed by equilibration with 95–5% buffer B in 1 min, and 5% buffer B for 10 min [27].

MS readings were acquired in positive ion mode using a Thermo Fisher Scientific Q Exactive hybrid quadrupole Orbitrap MS (Rockford, IL, USA). The instrument was equipped with a nanospray voltage of 1.5 kV and a source temperature of 250 ℃. Calibration was performed using a Proteo Mass LTQ/FT Hybrid ESI Pos. Mode Cal Mix (MS CAL5-1EA Supelco Sigma-Aldrich, St. Louis, MO, USA) as the external calibration solution. An internal calibration solution with a locked mass range of 445–12,003 was also employed. Operating in data-dependent acquisition mode, the MS initially scanned precursor ions in the mass range of m/z 400–1600 with a resolution of 70,000 (half-maximum full width at m/z 200). Subsequently, MS/MS scans were conducted for the ten highest intensity peaks of the 2+, 3+, 4+, and 5+ charged ions that exceeded the 16,000 ion threshold, at a resolution of 17,500. The Xcalibur 3.0.63 software (Thermo Scientific, Rockford, IL, USA) was used for data acquisition. All raw data were converted to mgf files and analyzed utilizing Proteome Discoverer 1.4 (Thermo Fisher Scientific, Rockford, IL, USA). The characterized data were compared with the genome of *G. lucidum* [28].

### 4.6. Statistical Analysis

We used TBtools v1.09876 to construct co-expression network modules. For cluster analysis and setting suitable thresholds for the samples, the Flash Clust toolkit in R v4.2.1 was employed. Using MetaboAnalyst 5.0, principal component analysis (PCA) and partial least squares regression-discriminant analysis (PLS-DA) was used to the normalized dataset that was collected using LC-MS. The variables were mean-centered and extracted using standard deviations before PCA and PLS-DA. The variables were extracted using standard deviations and centered around the mean before PCA and PLS-DA were performed. Proteins or metabolites were used as predictor factors in PLS-DA, whereas the response variables were the BS and control treatments (at all phases). The variables were weighted by the standard deviation and centered on the mean in order to guarantee equal variance. The PLS results were filtered using VIP scores to pinpoint crucial features. Next, a Student’s *t*-test was performed to identify metabolites, showing notable variances between the BS and control treatments across all phases. In terms of metabolic pathway assessment, pathways meeting the criteria of *p* < 0.05 in the hypergeometric test and pathway impact ≥ 0.1 were regarded as disrupted structures [29]. In order to reconstruct the metabolic pathways, a two-way analysis of variance (ANOVA) was utilized to compare the averages of each metabolite or protein among various treatments and stages. Phenotypical analyses were conducted using Student’s t-tests with a significance level of *p* < 0.05 through R software version 4.0.2 from Vienna, Austria, and the a‘gricolae’ package. It is important to mention that the experiments had at least three biological replicates (n ≥ 3).

Using the TBtools, cluster analysis of the samples was undertaken for WGCNA. The soft threshold, a critical parameter influencing the independence and average connectivity of the co-expression modules, was determined. Logarithms of the number of linked nodes (log(i)) and the probability of occurrence of each node (log(*p*(i))) were calculated in order to determine the ideal soft threshold value and meet the scale-free requirement for the adjacency function. The lowest value at which the correlation coefficient hits a plateau (or surpasses 0.8) was determined to be the soft threshold (β). In this investigation, β values of 9 and 8 were identified, promoting network scale-freeness. The modules were then divided into groups according to the clustering outcomes using the Dynamic Tree Cut technique, and each group was given a unique color. Similarity-sharing modules were merged, and each module’s eigenvalues were utilized to show the metabolite expression pattern for every sample. Additionally, a heatmap showing the example expression patterns was created.

## 5. Conclusions

Our findings provide a comprehensive overview of the proteome and metabolome during the development of *G. lucidum* from Luoshan, Shandong Province. A total of more than 2000 metabolites and 6000 proteins were identified, encompassing various categories such as glycerolipids, prenol lipids, fatty acyls, glycerophospholipids, steroids and steroid derivatives, purine nucleotides, amino acids, nucleotides, derivatives, triterpenoids, flavonoids, and phenolic acids. The traditional harvesting method for *G. lucidum* involves collecting the fruiting bodies before their spores are dispersed at maturity. Based on the results of our study, *G. lucidum* should be harvested at different stages, depending on the desired active ingredients. The best time for harvesting is during the BS if the goal is to obtain triterpenes and steroids. However, to obtain purine nucleotides, isoflavonoids, teridines, and derivatives, as well as phenanthrenes and derivatives, harvesting at the FS would be more suitable. Collectively, our findings offer novel and profound insights into the principal metabolites that influence the quality of *G. lucidum*, such as triterpenes and sterols, and simultaneously provide invaluable references for future breeding and cultivation endeavors. Furthermore, our study enhances the comprehension of the active ingredients and medicinal value of *G. lucidum* throughout its growth and development, providing a solid theoretical foundation for its utilization as a functional food.

## Figures and Tables

**Table 1:** Contents of amino acid in fruiting bodies of *G. lucidum*.

	Group	BS (mg/g)	PS (mg/g)	FS (mg/g)	MS (mg/g)
Amino Acid					
Asp		1.307 + 0.0036 a	1.1796 + 0.0009 b	1.0762 + 0.0006 c	0.7313 + 0.0023 d
Thr		0.8141 + 0.0015 a	0.7759 + 0.0014 b	0.7324 + 0.001 c	0.5369 + 0.0017 d
Ser		0.8563 + 0.0023 a	0.7623 + 0.001 b	0.6824 + 0.0004 c	0.4841 + 0.0011 d
Glu		2.1855 + 0.0031 a	2.0083 + 0.0029 b	1.5459 + 0.0015 c	0.9228 + 0.0014 d
Gly		0.7297 + 0.0017 a	0.6283 + 0.0004 b	0.5733 + 0.0001 c	0.3955 + 0.0005 d
Ala		0.8326 + 0.0016 a	0.7454 + 0.0022 b	0.6687 + 0.0009 c	0.4575 + 0.0005 d
Cys		0.1502 + 0.003 a	0.1375 + 0.0006 b	0.1339 + 0.0008 c	0.1186 + 0.0011 d
Val		0.7861 + 0.0026 a	0.6959 + 0.0026 b	0.6491 + 0.001 c	0.4561 + 0.0015 d
Met		3.6717 + 0.004 a	3.457 + 0.005 b	3.9251 + 0.0035 c	4.285 + 0.0046 d
Ile		0.5833 + 0.0027 a	0.5098 + 0.0009 b	0.501 + 0.0016 c	0.3549 + 0.0017 d
Leu		1.2311 + 0.0044 a	1.4158 + 0.0013 b	1.3719 + 0.0033 c	1.1637 + 0.0017 d
Tyr		0.3854 + 0.0021 a	0.3278 + 0.0014 b	0.2957 + 0.0026 c	0.1908 + 0.0008 d
Phe		0.6179 + 0.0025 a	0.6574 + 0.0006 b	0.5976 + 0.0018 c	0.4198 + 0.0016 d
Lys		0.7186 + 0.0035 a	0.6813 + 0.0019 b	0.6279 + 0.0011 c	0.3673 + 0.0219 d
His		0.273 + 0.0017 a	0.2654 + 0.0012 b	0.2286 + 0.001 c	0.1435 + 0.0006 d
Arg		0.7021 + 0.003 a	0.643 + 0.0014 b	0.5532 + 0.0032 c	0.3115 + 0.0027 d
pro		0.6063 + 0.0046 a	0.5379 + 0.003 b	0.4886 + 0.0038 c	0.3205 + 0.0029 d

Data are mean ± standard deviation of percentages (*n* = 6); different lowercase letters in the same row indicate significant differences (*p* < 0.05).

**Table 2:** Detailed amounts of metabolite differences at different growth stages of *G. lucidum*.

Metabolite Category	BS–PS		BS–FS		BS–MS	
	Up	Down	Up	Down	Up	Down
Fatty Acyls	27	4	19	3	22	5
Benzene and substituted derivatives	18	14	24	11	30	12
Carboxylic acids and derivatives	15	17	34	10	36	7
Organooxygen compounds	9	12	5	6	38	8
Glycerolipids	8	6	2	15	8	14
Prenol lipids	7	13	10	12	16	10
Organonitrogen compounds	6	12	5	6	4	8
Flavonoids	5	4	3	4	5	4
Purine nucleosides	4	8	9	4	10	4
Others	135	214	214	118	231	122

## Data Availability

The raw data supporting the conclusions of this article will be made available by the authors upon request.

## Supplementary Materials

The following supporting information can be downloaded at: https://www.mdpi.com/article/10.3390/molecules29112591/s1, Supplementary File S1: Determination of nutrient composition and micro- and macro-elements, Supplementary File S2: LC-MS analysis, Supplementary Figures: Figure S1: Molecular identification results, Figure S2: The heatmap of metabolite, Figure S3: Proteome WGCNA analysis (A) Analysis of network topology for various soft-thresholding powers. The left panel shows the scale-free fit index (y-axis) as a function of the soft-thresholding power (x-axis). The right panel displays the mean connectivity (degree, y-axis) as a function of the soft-thresholding power (x-axis); (B) Clustering dendrogram of proteomics, with dissimilarity based on topological overlap, together with assigned module colors; (C) Clustering dendrogram of samples with trait heatmap; (D) Module-trait associations: Each row corresponds to a module eigengene and each column to a trait. Each cell contains the corresponding correlation and p-value. The table is color-coded by correlation according to the color, Figure S4: The heatmap of triterpenoid, Table S1: Metabolome Classification Enumeration Table, Table S2: The Table of KEGG, Table S3: The Table of Metabolism, Table S4: The Table of Proteomics.
