# Supplementary material for: Changes of Active Substances in Ganoderma lucidum during Different Growth Periods and Analysis of Their Molecular Mechanism
Source: Molecules. 2024 May 31;29(11):2591. doi: 10.3390/molecules29112591 (PMC11173900; doi:10.3390/molecules29112591)
Supplement: Supplementary file 1 [file molecules-29-02591-s001.zip › supplement Figure.pdf]

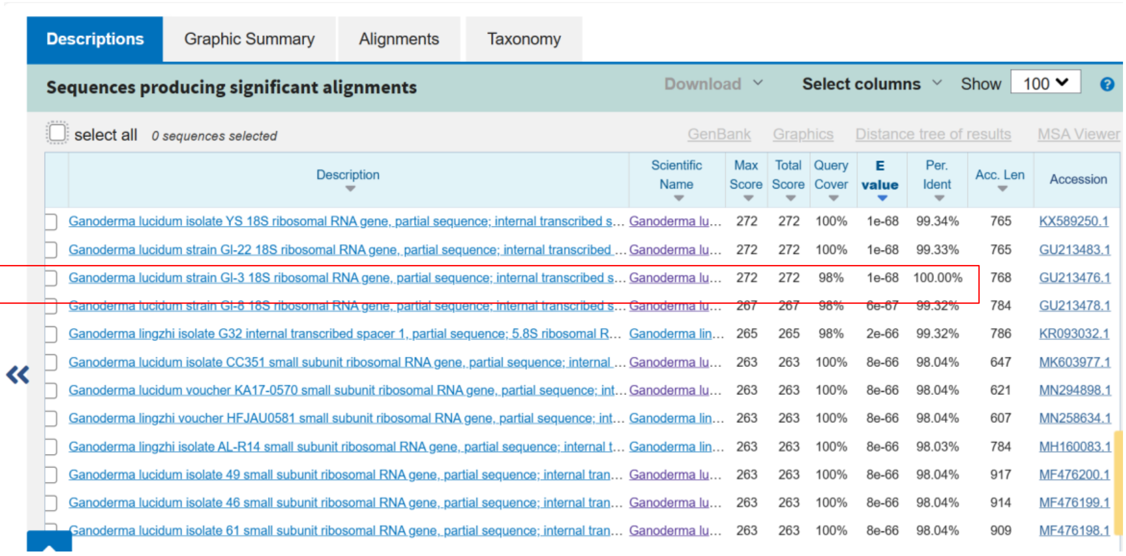

Description	Scientific Name	Max Score	Total Score	Query Cover	E value	Per. Ident	Acc. Len	Accession
Ganoderma lucidum isolate YS 18S ribosomal RNA gene, partial sequence; internal transcribed s…	Ganoderma lu…	272	272	100%	1e-68	99.34%	765	KX589250.1
Ganoderma lucidum strain GI-22 18S ribosomal RNA gene, partial sequence; internal transcribed …	Ganoderma lu…	272	272	100%	1e-68	99.33%	765	GU213483.1
Ganoderma lucidum strain GI-3 18S ribosomal RNA gene, partial sequence; internal transcribed s…	Ganoderma lu…	272	272	98%	1e-68	100.00%	768	GU213476.1
Ganoderma lucidum strain GI-8 18S ribosomal RNA gene, partial sequence; internal transcribed s…	Ganoderma lu…	267	267	98%	6e-67	99.32%	784	GU213478.1
Ganoderma lingzhi isolate G32 internal transcribed spacer 1, partial sequence; 5.8S ribosomal R…	Ganoderma lin…	265	265	98%	2e-66	99.32%	786	KR093032.1
Ganoderma lucidum isolate CC351 small subunit ribosomal RNA gene, partial sequence; internal …	Ganoderma lu…	263	263	100%	8e-66	98.04%	647	MK603977.1
Ganoderma lucidum voucher KA17-0570 small subunit ribosomal RNA gene, partial sequence; int…	Ganoderma lu…	263	263	100%	8e-66	98.04%	621	MN294898.1
Ganoderma lingzhi voucher HFJAU0581 small subunit ribosomal RNA gene, partial sequence; int…	Ganoderma lin…	263	263	100%	8e-66	98.04%	607	MN258634.1
Ganoderma lingzhi isolate AL-R14 small subunit ribosomal RNA gene, partial sequence; internal t…	Ganoderma lin…	263	263	100%	8e-66	98.03%	784	MH160083.1
Ganoderma lucidum isolate 49 small subunit ribosomal RNA gene, partial sequence; internal tran…	Ganoderma lu…	263	263	100%	8e-66	98.04%	917	MF476200.1
Ganoderma lucidum isolate 46 small subunit ribosomal RNA gene, partial sequence; internal tran…	Ganoderma lu…	263	263	100%	8e-66	98.04%	914	MF476199.1
Ganoderma lucidum isolate 61 small subunit ribosomal RNA gene, partial sequence; internal tran…	Ganoderma lu…	263	263	100%	8e-66	98.04%	909	MF476198.1

Figure. S1 Molecular identification results

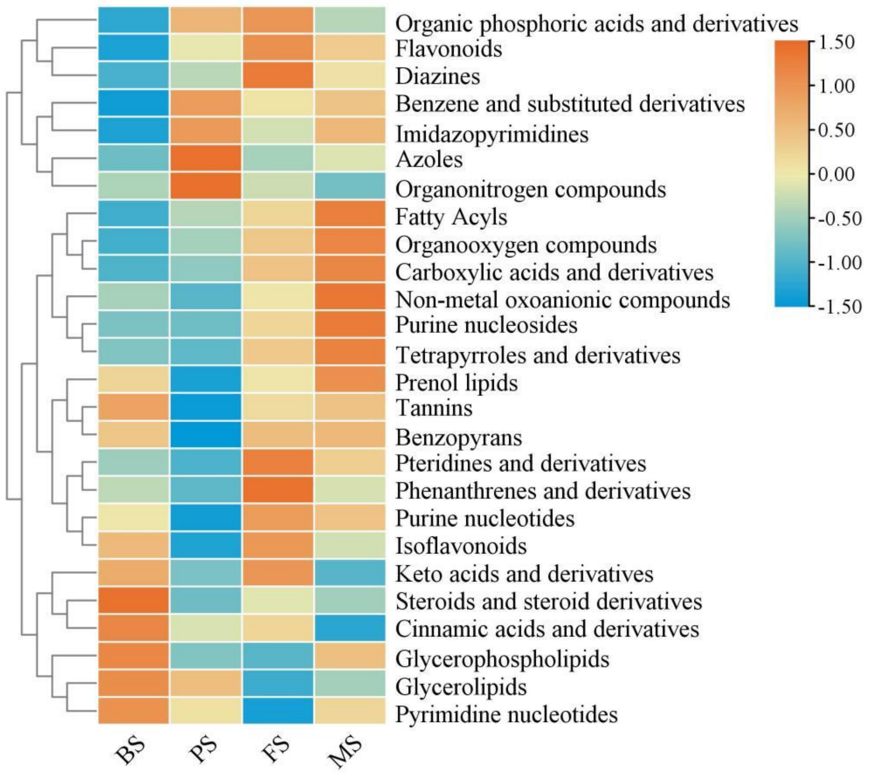

Figure. S2 The heatmap of metabolite

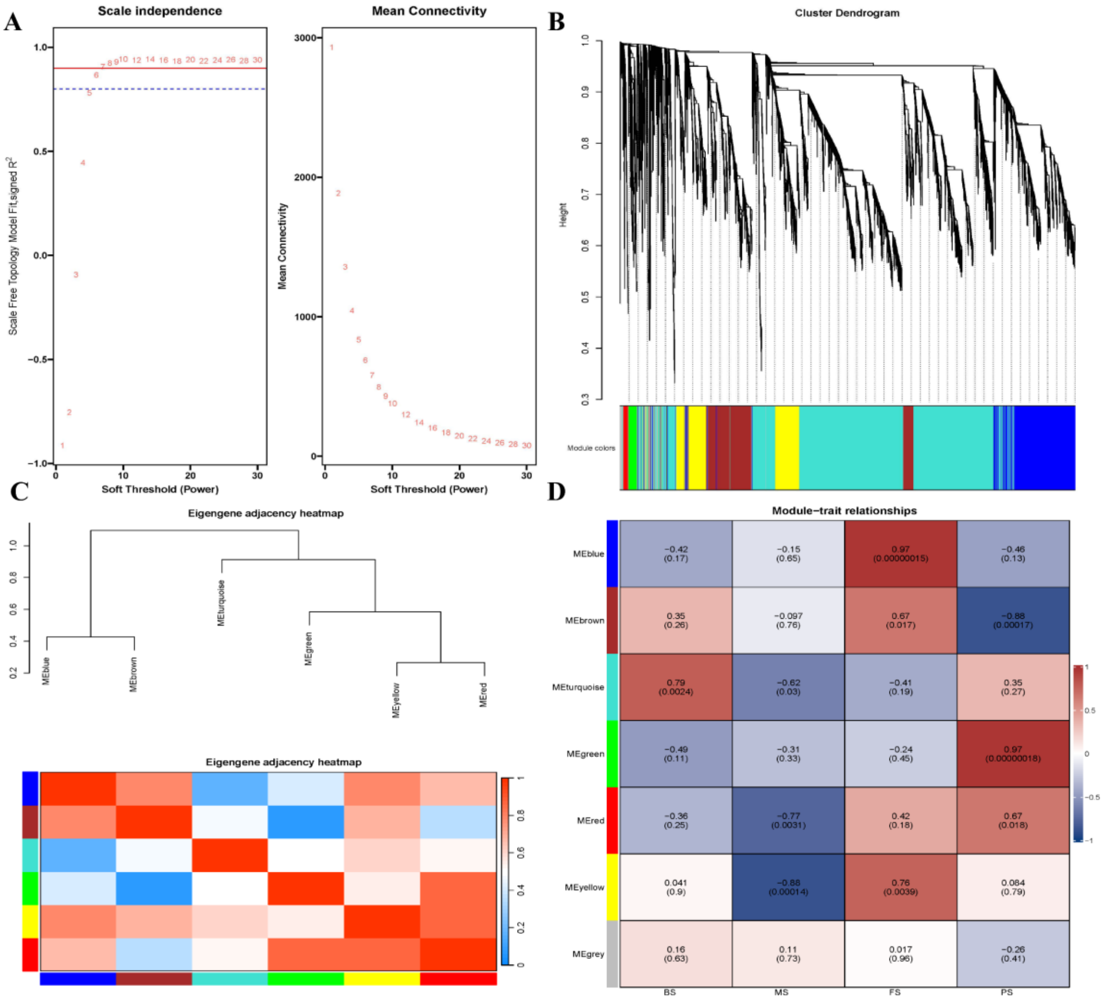

Figure. S3 Proteome WGCNA analysis (A) Analysis of network topology for various soft-thresholding powers. The left panel shows the scale-free fit index (y-axis) as a function of the soft-thresholding power (x-axis). The right panel displays the mean connectivity (degree, y-axis) as a function of the soft-thresholding power (x-axis); (B) Clustering dendrogram of proteomics , with dissimilarity based on topological overlap, together with assigned module colors; (C) Clustering dendrogram of samples with trait heatmap; (D) Module-trait associations: Each row corresponds to a module eigengene and each column to a trait. Each cell contains the corresponding correlation and p-value. The table is color-coded by correlation according to the color.

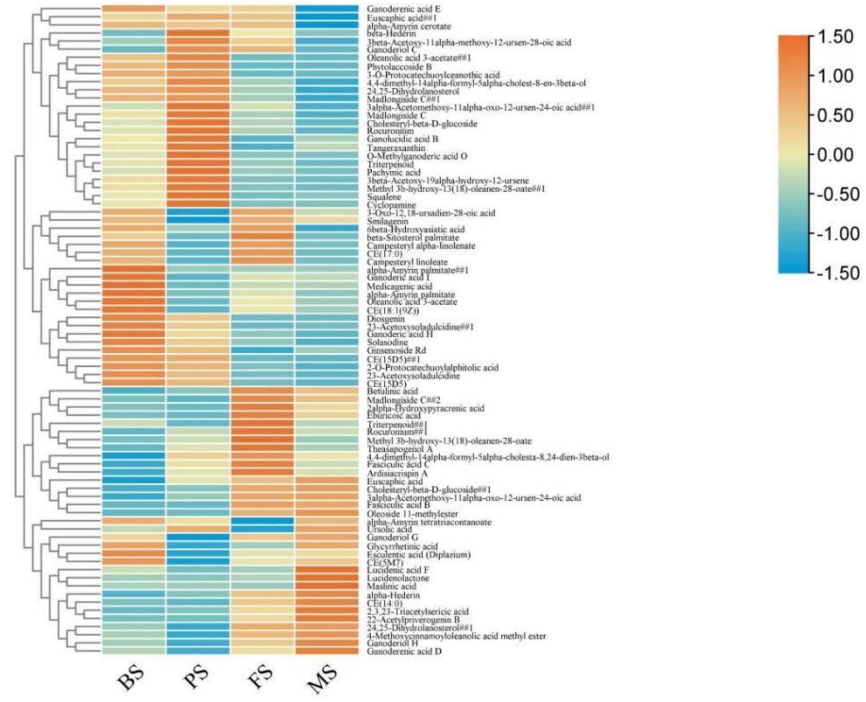

Figure. S4 The heatmap of triterpenoid
