# Supplementary material for: Changes of Active Substances in Ganoderma lucidum during Different Growth Periods and Analysis of Their Molecular Mechanism
Source: Molecules. 2024 May 31;29(11):2591. doi: 10.3390/molecules29112591 (PMC11173900; doi:10.3390/molecules29112591)
Supplement: Supplementary file 1 [file molecules-29-02591-s001.zip › Supplement S1.pdf]

molecules

MDPI

Article

Changes of active substances in *Ganoderma lucidum* during different growth periods and analysis of their molecular mechanism

Xusheng Gao[1†], Huimin Huo[1†], Haiying Bao[1*], Jialu Wang[2], Dan Gao[2*]

[1] College of Traditional Chinese Medicine and Key Laboratory of Edible Fungi Resources and Utilization, Min-istry of Agriculture and Rural Affairs, Jilin Agricultural University, Changchun 130118, China, gaoxusheng@o.cnu.ac.kr (X.S.G.); huohuimin1216@163.com (H.H.M.); baohaiying@jlau.edu.cn (H.Y.B.)
[2] Institute of Chinese Materia Medica, China Academy of Chinese Medical Sciences, Beijing 100700, China, JialuMarco2019@outlook.com (J.L.W.); dgao@icmm.ac.cn (D.G.)

* Correspondence: baohaiying@jlau.edu.cn (H.Y.B.); dgao@icmm.ac.cn (D.G.)

† These authors have contributed equally to this work.

1. Determination of nutrient composition and micro- and macro-elements

The determination of polysaccharide content involves precise weighing of a 2 g sample powder, followed by reflux extraction in 100 mL of distilled water for 3 hours. The mixture is then hot filtered at a pressure of 0.07 ~ 0.08 MPa, with subsequent washing of the filter and residue using distilled water. This extraction process is repeated three times. The combined extracts are concentrated to 30 mL using a water bath and then mixed with 75 mL of anhydrous ethanol. After overnight incubation at 4 °C, the mixture is centrifuged at 2432 g for 15 minutes. The resulting precipitate is dissolved in distilled water and the volume is adjusted to 100 mL. The polysaccharide content is determined by measuring the absorbance at 490 nm using the phenol sulfuric acid method. A standard curve is constructed using glucose solutions with different mass concentrations to calculate the polysaccharide content in each sample.

For the determination of flavonoid content, a 2 g sample powder is finely weighed and then subjected to backflow extraction in 100 mL of 95% ethyl alcohol for 5 hours. The mixture is then filtered at a pressure of 0.07 ~ 0.08 MPa, followed by washing of the filter and residue. The extract is concentrated on a water bath to a volume of 5 ~ 10 mL and washed with 60% ethyl alcohol. The absorbance at 504 nm is measured using the nitrite-aluminum nitrate method, and the flavonoid content in each sample is calculated using standard curves constructed with rutin solutions of different mass concentrations.

The content of triterpenes and sterols is determined by ultrasonic extraction of 2 g of sample powder in a test tube with a plug using 30 mL of 75% ethanol for 3.5 hours at a power of 100 W and a frequency of 50 Hz. The mixture is then filtered at a pressure of 0.07 ~ 0.08 MPa, with subsequent washing of the filter and residue using 75% ethanol. The filtrate is transferred to a 50 mL volumetric bottle and the absorbance at 550 nm is measured using the vanilla-perchloric acid method. The contents of triterpenes and sterols in the samples are calculated using standard curves constructed with oleanolic acid solutions of different mass concentrations.

To determine the polyphenol content, a 2 g sample powder is accurately weighed and subjected to ultrasonic extraction in methanol for 3.5 hours at a power of 100 W and a frequency of 50 Hz. The extraction liquid is collected after filtration and the volume is adjusted to 50 mL. The absorbance at 765 nm is measured using the Folin phenol method.

The polyphenol content in the sample is calculated using standard curves constructed with gallic acid solutions of different mass concentrations.

For the determination of crude protein content, a 0.1 g sample powder is accurately weighed and analyzed using an automatic Kjeldahl nitrogen analyzer. Similarly, the content of macro elements, trace elements, and heavy metals in the sample is determined by using an inductively coupled plasma mass spectrometer after accurately weighing a 0.1 g sample powder.

2.Elemental Determination

2.1Standard solution, internal standard solution preparation

Dilute the mixed standard solution with 3% nitric acid to formulate the concentration of 0.01, 0.05, 0.1, 0.5, 1, 5, 10, 50, 100, 500 ng/mL The solutions were prepared in concentrations of 0.01, 0.05, 0.1, 0.5, 1, 5, 10, 50, 100, 500 ng/mL. The standard solution of Hg element was diluted with 3% nitric acid, and was prepared as A gradient dilution of the Hg elemental standard solution with 3% nitric acid was used to make solutions at concentrations of 0.05, 0.1, 0.2, 0.5, 1, 2 ng/mL.

2.2 Preparation of test solution and blank control solution

Weigh 0.3 g of the sample powder (sieve No.3), put it in a PTFE high-pressure microwave digestion tank, add 5 mL of nitric acid, mix well, and then transfer it into the microwave digestion instrument after 2 h of resting. The dissolution was carried out according to the set procedure: 10 min ramp-up at 800 W, maintained for 5 min, 15 min ramp-up at 1600 W, maintained for 25 min, and then removed from the dissolution tank and cooled to room temperature under pressure. After completion of the digestion, remove the digestion tank, cool to room temperature under pressure, transfer the digestion solution to a 25 mL volumetric flask, and wash with ultrapure water. Wash the tank with ultrapure water for three times, and then combine the washed solution into a volumetric flask. Add 100 μL of Au internal standard solution into the volumetric flask, add ultrapure water to 25 mL, shake well.

Shake well, the test solution was obtained. Prepare blank control solution in the same way.

2.3 Examination of linearity and detection limit

The standard solutions of different concentrations were measured according to the above conditions, and the linear regression of the measured response value (Y) on the concentration (X) was carried out to obtain the regression equation, and the blank control solution was measured for 10 times to calculate the detection limits of each element, and the linear ranges of the elements were 0.01-500 ng/mL, and the correlation coefficients of the linear equations of the elements were greater than 0.999 9, indicating that the linear relationship and detection limit of the article are not significant. The correlation coefficients of the linear equations were all greater than 0.9999, indicating that the linear relationships of the elements were good under the conditions. The correlation coefficients of the linear equations of the elements were all greater than 0.999 9, indicating that the linear relationships of the elements were good under the conditions.

2.4 Determination of amino acids

The determination of amino acid content was conducted using an automatic amino acid analyzer according to the GB5009.124-2016 standard. Samples were hydrolyzed in 6 N HCl at 110°C for 24 hours, then centrifuged and filtered to remove insoluble materials. The hydrolysates were then subjected to the automatic amino acid analyzer for separation and quantification, using pre-column derivatization to ensure accurate quantification of

amino acids. Proline was quantified with a detection wavelength of 440 nm, while other amino acids were detected at 570 nm.

3.Methodological Examination

3.1Precision test

The mixed standard solutions were aspirated and measured 6 times, and the RSD was calculated. The results showed that the RSD ranged from 0.5% to 2.08%, which indicated that the precision of the instrument was good.

3.2 Stability test

The stability test was carried out by taking the test solution every 30 min for 6 times. The RSD was 1.22%-3.01%, indicating that the test solution was stable within 180 min. The RSD was 1.22%-3.01%, indicating that the test solution was stable within 180 min.

3.3 Sample recovery test

Appropriate amount of sample powder was weighed precisely and added into 0.1 mL of standard solution, then measured and calculated the sample recovery. The recoveries of each element ranged from 94.07% to 108.58%.

The RSDs of the three recoveries were 2.35%~4.67%, indicating that the method is accurate and reliable.2.1. Subsection
