# Supplementary material for: Changes of Active Substances in Ganoderma lucidum during Different Growth Periods and Analysis of Their Molecular Mechanism
Source: Molecules. 2024 May 31;29(11):2591. doi: 10.3390/molecules29112591 (PMC11173900; doi:10.3390/molecules29112591)
Supplement: Supplementary file 1 [file molecules-29-02591-s001.zip › supplement S2.pdf]

molecules

MDPI

Article

Changes of active substances in *Ganoderma lucidum* during different growth periods and analysis of their molecular mechanism

Xusheng Gao[1†], Huimin Huo[1†], Haiying Bao[1*], Jialu Wang[2], Dan Gao[2*]

[1] College of Traditional Chinese Medicine and Key Laboratory of Edible Fungi Resources and Utilization, Ministry of Agriculture and Rural Affairs, Jilin Agricultural University, Changchun 130118, China, gaoxusheng@o.cnu.ac.kr (X.S.G.); huohuimin1216@163.com (H.H.M.); baohaiying@jlau.edu.cn (H.Y.B.)
[2] Institute of Chinese Materia Medica, China Academy of Chinese Medical Sciences, Beijing 100700, China, JialuMarco2019@outlook.com (J.L.W.); dgao@icmm.ac.cn (D.G.)
* Correspondence: baohaiying@jlau.edu.cn (H.Y.B.); dgao@icmm.ac.cn (D.G.)
† These authors have contributed equally to this work.

1.LC-MS analysis

1.1 Metabolomic analysis

Liquid Chromatography-Mass Spectrometry (LC-MS) analysis was performed using the UHPLC-Q Exactive HF-X system. Samples were separated on a Waters ACQUITY UPLC HSS T3 Column (1.8 μm, 100 mm × 2.1 mm). The mobile phase consisted of a water-acetonitrile (95:5, v/v) mixture with 0.1% formic acid (A) and an acetonitrile-isopropanol-water (47.5:47.5:5, v/v/v) mixture with 0.1% formic acid (B). The gradient elution followed this program: 0–24.5% B in 3.5 minutes, 24.5–65% B in 1.5 minutes, 65–100% B in 0.5 minutes, 100% B in 1.9 minutes, 100–51.5% B in 0.2 minutes, 51.5–0% B in 0.2 minutes, and 0% B in 2.2 minutes. The operational parameters included a 2 μL injection volume, a flow rate of 0.4 mL/min, and a column temperature of 40 ℃. Electrospray ionization (ESI) was employed in both positive and negative modes for MS data acquisition. The MS conditions were as follows: a scan range (m/z) of 70–1050 Da, a sheath gas flow rate of 50 arb, an aux gas flow rate of 13 arb, a heater temperature of 425 ℃, a capillary temperature of 325 ℃, a spray voltage of 3500 V (+) and 3500 V (), and an S-Lens RF Level of 50. The fragments were subjected to a normalized collision energy of 27, 29, and 32 eV. Peak intensity refinement involved filtering low mass peaks, imputing missing values, normalizing data, evaluating the Relative Standard Deviation (RSD) of quality control samples, and converting the data. Missing value imputation was performed using the Random Forest algorithm's automatic selection option in the software. Quantification was carried out using a Q Exactive mass spectrometer in both full scan and MS2 modes, with a resolution of 60,000 for full MS scan and 7,500 for MS2 scan.

1.2 Data processing and metabolite identification

The raw data files generated by LC-MS were processed using the Compound Discoverer 3.1 (CD3.1, Thermo Fisher) to perform peak alignment, peak picking, and quantitation for each metabolite. The main parameters were set as follows: retention time tolerance, 0.2 minutes; actual mass tolerance, 5ppm; signal intensity tolerance, 30%; signal/noise ratio, 3; and minimum intensity, 100,000. After that, peak intensities were normalized to the total spectral intensity. The normalized data was used to predict the molecular formula based on additive ions, molecular ion peaks and fragment ions. And then peaks were matched with the mzCloud (https://www.mzcloud.org/)，mzVault and

MassList database to obtain the accurate qualitative and relative quantitative results. Statistical analyses were performed using the statistical software R (R version R-3.4.3), Python (Python 2.7.6 version) and CentOS (CentOS release 6.6). When data were not normally distributed, normal transformations were attempted using of area normalization method.
